# MiR-21-5p regulates extracellular matrix degradation and angiogenesis in TMJOA by targeting Spry1

**DOI:** 10.1186/s13075-020-2145-y

**Published:** 2020-05-01

**Authors:** Shixing Ma, Aobo Zhang, Xiaole Li, Shizhou Zhang, Shaopeng Liu, Haoming Zhao, Shichao Wu, Lei Chen, Chuan Ma, Huaqiang Zhao

**Affiliations:** 1 Shandong Provincial Key Laboratory of Oral Tissue Regeneration & Department of Oral and Maxillofacial Surgery, School of Stomatology, Shandong University, Number 44, Wen Hua Xi Lu, Jinan City, 250012 Shandong Province China; 2 Shandong Provincial Key Laboratory of Oral Tissue Regeneration & Department of Orthodontics, School of Stomatology, Shandong University, Jinan, Shandong China; 3 Ningbo Stomatology Hospital, Number 287, Jie Fang Nan Lu, Ningbo, 315010 Zhejiang Province China; 4 The Institute for Tissue Engineering and Regenerative, Liaocheng People’s Hospital, Liaocheng, Shandong China

**Keywords:** miR-21-5p, Spry1, TMJOA, VEGF, MMP-13, ERK-MAPK signalling pathway

## Abstract

**Background:**

Due to the lack of research on the pathological mechanism of temporomandibular joint osteoarthritis (TMJOA), there are few effective treatment measures in the clinic. In recent years, microRNAs (miRs) have been demonstrated to play an important role in the pathogenesis of osteoarthritis (OA) by regulating a variety of target genes, and the latest evidence shows that miR-21-5p is specifically overexpressed in OA. The purpose of this project was to clarify whether miR-21-5p can regulate the TMJOA process by targeting Spry1.

**Methods:**

TMJOA was induced by a unilateral anterior crossbite (UAC) model, and the effect of miR-21-5p knockout on TMJOA was evaluated by toluidine blue (TB), immunohistochemical (IHC) staining, Western blotting (WB) and RT-qPCR. Primary mouse condylar chondrocytes (MCCs) were isolated, cultured and transfected with a series of mimics, inhibitors, siRNA-Spry1 or cDNA Spry1. WB, RT-qPCR, IHC and TB were used to detect the effect of miR-21-5p and its target gene Spry1 on the expression of MMP-13, VEGF and p-ERK1/2 in TMJOA. The effect of miR-21-5p on angiogenesis was evaluated by chick embryo chorioallantoic membrane (CAM) assay and WB.

**Results:**

In the UAC model, the cartilage thickness and extracellular matrix of miR-21-5p knockout mice were less damaged, and miR-21-5p and UAC model were shown to affect the expression of Spry1, IL-1β, MMP-13, and VEGF. Luciferase experiments confirmed that Spry1 was the direct target of miR-21-5p. The expression levels of Spry1, MMP-13, VEGF and p-ERK1/2 in MCCs transfected with miR-21-5p mimic were higher than those in the inhibitor group. Under the simulated inflammatory environment of IL-1β, the expression levels of MMP-13, VEGF and p-ERK1/2 were positively correlated with miR-21-5p, while Spry1 was negatively correlated with miR-21-5p. Inhibition of miR-21-5p expression and overexpression of Spry1 enhanced the inhibition of MMP-13, VEGF and p-ERK1/2 expression. MiR-21-5p had a significant role in promoting angiogenesis in the chick embryo CAM assay, and this role was clearly mediated by the ERK-MAPK signalling pathway.

**Conclusion:**

This study verified that miR-21-5p can promote the process of TMJOA by targeting Spry1, which provides a new direction for future research on the treatment of this disease.

## Background

Temporomandibular disorders (TMD) are a complex and controversial disease [1]. Temporomandibular joint osteoarthritis (TMJOA) is characterized by destruction and loss of the articular cartilage matrix, subchondral osteosclerosis, synovitis and osteophyte, which is one of the most serious diseases in the TMD classification [2]. The temporomandibular joint (TMJ) is the only joint composed of fibrocartilage, so it is different from other joints composed of hyaline cartilage in structure and function. Degenerative changes in articular cartilage in patients with TMJOA are generally considered to be due to self-reconstruction after dysfunction of the joint structure. However, it is still difficult to explain the pathophysiological mechanism that leads to the progression of this disease. Further studies of extracellular regulatory factors and intracellular signalling mechanisms that regulate articular cartilage homeostasis have helped to discover new therapeutic targets for osteoarthritis [3].

Due to their specific anatomy and location, chondrocytes survive in an environment with a lack of blood vessels and hypoxia and maintain tissue homeostasis. Blood vessels originating in the subchondral bone can enter the cartilage microenvironment which lacks blood vessels through further growth and breaking through the tide mark, thus stimulating the ossification of articular cartilage and leading to OA. In the subchondral bone of OA, the severity of structural changes is positively correlated with the expression of vascular endothelial growth factor (VEGF) secreted by chondrocytes [4]. In addition, new research shows that VEGF can play a key role in inducing chondrocyte apoptosis and cartilage degeneration of the TMJ by increasing the expression of matrix metalloproteinases (MMPs) [5]. MMPs are a family of major enzymes that degrade extracellular collagen and aggrecan (ACAN) in OA. In chondrocytes, VEGF can promote the secretion of MMP-1, MMP-3 and especially MMP-13 [6, 7]. In addition, the activation of the ERK-MAPK signalling pathway has been confirmed to promote the secretion of MMPs in chondrocytes and osteoblasts, thus promoting the development of OA [8]. Therefore, abnormal expression of VEGF and activation of the ERK-MAPK signalling pathway are important factors leading to angiogenesis and extracellular matrix degradation of TMJOA.

MicroRNAs are a class of endogenous non-coding RNAs 19–25 nucleotides in length that can regulate the expression of target genes through complementary pairing with the target gene 3′-untranslated region (3′ UTR), resulting in mRNA degradation or translational inhibition [9]. Recent studies have confirmed that microRNAs can participate in cartilage formation, cartilage degradation and OA development by regulating various cell processes, such as apoptosis, cell differentiation, proliferation and matrix remodelling [10–12]. Thus, microRNAs play an important role in the pathogenesis of OA. Through the use of Gene Expression Omnibus (GEO) analysis and the detection of miR-21-5p expression in OA patient cartilage, it has been found that the expression of miR-21-5p in OA is significantly upregulated [13, 14]. In addition, the latest study found that miR-21-5p can promote the degradation of extracellular matrix by regulating its target gene fibroblast growth factor 18 (FGF18) and suggested that miR-21-5p may be a new target for OA treatment [13]. It can be considered that miR-21-5p, as a new target closely related to OA, is likely to play a crucial role in the pathogenesis and progression of TMJOA.

Sproutys (Sprys) are negative regulators of ERK1/2 activation induced by RTK [15, 16], and Spry1 is one of the four identified mammalian Spry homologues (Spry1, 2, 3, 4) [17]. Studies have shown that the main function of Spry1 is to inhibit the Ras/Raf/ERK signalling pathway, and the absence of Spry1 activates phosphorylation of the ERK-MAPK signalling pathway [15, 18]. The protein encoded by the Spry1 gene also exhibits this inhibitory function under the stimulation of a series of growth factors, and VEGF is one of the major stimulators of the Spry1 protein [16]. It can be seen from the above research background that the abnormal expression of VEGF and MMPs and activation of the ERK-MAPK signalling pathway play a critical role in the pathogenesis of TMJOA, so Spry1 is likely to participate in the inflammatory destruction process of TMJOA. Interestingly, we found that Spry1 was the direct target of miR-21-5p by bioinformatics analysis and dual-luciferase assay. Therefore, we hypothesized that miR-21-5p could upregulate the secretion of VEGF and MMPs and activate the ERK-MAPK signalling pathway by targeting Spry1 to promote the process of TMJOA.

The purpose of this experiment was to explore whether miR-21-5p can promote the TMJOA process by targeting Spry1. This experiment was the first to investigate the effects of miR-21-5p and its target gene Spry1 on the degradation and angiogenesis of TMJ. We found that miR-21-5p knockout (KO) mice had a lower degree of TMJ cartilage destruction than the wild-type (WT) mice in the established TMJOA model. In vitro results showed that miR-21-5p could inhibit the expression of Spry1, resulting in the upregulation of MMP-13, VEGF expression and phosphorylation of ERK-MAPK signalling pathway components.

## Methods

### Animals and dental operation

All procedures and protocols were approved by the Ethics Committee of Shandong University. Twenty WT male C57BL/6N mice (6–8 weeks of age, weight 18–21 g) were obtained from the Animal Centre of Shandong University (Jinan, China). Twenty miR-21-5p^**−/−**^ C57BL/6 knockout (miR-21-5p KO) mice were bred from miR-21-5p KO mice (two males, two females) purchased from Southeast University (Nanjing, China). KO mouse genotyping results are listed in Supplementary 1. WT mice were randomly divided into a control group (WT-NC) and unilateral anterior crossbite group (WT-UAC). MiR-21-5p KO mice were randomly divided into a control group (KO-NC) and unilateral anterior crossbite group (KO-UAC). Metal tubes were placed in the left maxillary incisor and mandibular incisor of the intervention group, and the intervention time was 3 weeks. The operation steps of the UAC model were performed as described [19]. Mice in the NC groups underwent all procedures, but no metal tube was bonded. All mice received a standardized diet.

### Histology and immunohistochemistry (IHC) staining

After 3 weeks, six mice (12 TMJs) were randomly selected from each group for toluidine blue (TB) and IHC, and four mice (8 TMJs) were used for Western blotting (WB) and RT-qPCR. After fixation in paraformaldehyde, the samples were decalcified in EDTA for 4 weeks, then embedded in paraffin, and 5-μm-thick sections were prepared. After deparaffinization, the sections were stained with TB (Sigma-Aldrich, Poole, UK). Collagen fibres and ACAN are the main extracellular matrix components of cartilage and can be stained blue-violet by the basic dye TB. Image-Pro Plus 6.0 software was used to quantify the average optical density (AOD) values of the stained area. Blue-stained areas were selected as the uniform standard for evaluating all images. Each image was analysed to obtain the integrated optical density (IOD) and the area of the pixel (AREA) of the tissue. Finally, the AOD (AOD = IOD/AREA) was determined. Using the image scale (50 μm) as the standard, the cartilage thickness (millimetres) at 5 positions was measured for each image, and the average value was obtained.

For IHC, the sections were dewaxed and 0.1% trypsin was applied to each section for 10 min at 37 °C, and the endogenous peroxidase activity of the sections was quenched using 3% H_2_O_2_ for 10 min. Then, sections were blocked with 5% bovine serum albumin (BSA) for 30 min and incubated with primary antibody against aggrecan (ACAN) (1:100; Abcam, MA, USA) at 4 °C overnight. Then, the sections were incubated with the secondary antibody and horseradish peroxidase-conjugated avidin (BOSTER, SA1022, China). For IHC staining, the percentages of positively stained cells were determined. The AOD of the positive area was measured.

### Target prediction and luciferase reporter assay

To predict the target relationship between miR-21-5p and Spry1, a miRNA prediction software package, miRanda (http://www.microrna.org/microrna/home.do), which is widely used to confirm the theoretical target genes of miRNAs, was used in this study. To further verify the reliability of the target gene prediction, MCCs were co-transfected with a luciferase reporter containing the target fragment (Spry1-3′ UTR-WT) or with a vector carrying the mutant-type 3′ UTR of Spry1 (Spry1-3′ UTR-MUT) and with miR-21-5p mimic or the corresponding negative control (NC). The amplified primers sequences of 3′ UTR of Spry1 were as follows: Spry1 sense: CTT TGT GCC TAC CCT GCT TGC TCT GCT ACC and Spry1 anti-sense: AGG GCG GTG GGT CCA GTC GTA ACA GC. After 48 h, the luciferase activity was calculated using the dual-luciferase reporter assay system according to the manufacturer’s instructions (Promega, Madison, WI, USA).

### Culture and identification of condylar chondrocytes

MCCs were isolated from twenty mice (2-week-old, male, 5 g). Samples were minced into pieces of less than 1 mm^3^ with microsurgical scissors, followed by digestion at 37 °C with 3 mg/ml dispase (Sigma-Aldrich, USA) 1× and 2.5 mg/ml collagenase type II in DMEM for 2 h with stirring every 20 min. The single-cell suspensions were cultured (5% CO_2_, 37 °C) in 79% DMEM supplemented with 20% FBS, 100 mg/ml streptomycin and 100 mg/ml penicillin solution for 5 to 7 days before use. The cells were digested with trypsin-EDTA and passaged into a T25 culture flask for subsequent experiments. Additionally, the chondrocytes were validated through immunocytochemical identification of type II collagen (Supplementary 2).

### Cell transfection

MCCs were cultured to 85% confluence in 6-well plates. Transfection of MCCs using Lipofectamine 2000 (Thermo Fisher, USA, catalogue no. 11668-019, 4 μl/well) was performed with the synthetic precursors of miR-21-5p called miR-21-5p mimic, mimic negative control (mimic NC), miR-21-5p inhibitor and inhibitor negative control (inhibitor NC) (Genepharma, Shanghai, China). The cells were transfected with miR-21-5p mimic or inhibitor to a final concentration of 50 nM with Lipofectamine 2000 according to the manufacturer’s protocols. After 6 h, the medium was replaced with DMEM supplemented with 20% FBS. After 48 h, RNA and protein were extracted and analysed.

The vector for Spry1 overexpression was GV141, the enzyme digestion cloning site was XhoI/KpnI, and the empty vector was used as the control (GeneChem, Shanghai, China). The target gene sequence for chemical synthesis and the primer sequence for identification of the recombinant plasmid are listed in Supplementary 3. MCCs were transfected with the aforementioned vectors using Lipofectamine 2000. For Spry1 knockdown, chondrocytes were transfected with small interference RNA of mouse Spry1 (si-Spry1) to suppress Spry1 expression. Chondrocytes were transfected with Spry1-specific small interfering RNA (si-Spry1) or negative control siRNA (si-NC) (GeneChem, Shanghai, China) using Lipofectamine 2000. After transfection, the chondrocytes were incubated with 10 ng/ml IL-1β (R&D systems, Abingdon, UK) for another 24 h. The transfection efficiency was determined using Western blotting. The sequences of the mimic, inhibitor and siRNA are listed in Table 1.

**Table 1:** Transfection sequences for miR-21-5p mimic, miR-21-5p inhibitor and siRNA-Spry1

Gene	Sequence (5′-3′)
**miR-21-5p mimic**	**UAGCUUAUCAGAC UGAUGUUGA**
**miR-21-5p inhibitor**	**UAGCUUAUCAGACUGAUGUUGA**
**siRNA-Spry1**	**CCCAGAATGTTGACAGCTGCCTCTT**

*miR-21-5p* microRNA-21-5p, *siRNA* small interfering RNA

### Western blotting

Condylar cartilage was incubated in liquid nitrogen and ground to a fine powder. MCCs were collected from plates and washed with DPBS. Tissue and cells were lysed using RIPA with 1% phenylmethanesulfonyl fluoride (PMSF) (Beyotime, China) followed by centrifugation at 12,000 rpm for 15 min at 4 °C, and the resulting supernatants were quantified by the bicinchoninic acid (BCA) assay. A 10% sodium dodecyl sulfate separation gel and a concentration gel were prepared. Transfer of the proteins to nitrocellulose membranes was carried out at 60 V for 1 h and 120 V for 0.5 h. The polyvinylidene difluoride membranes (Millipore, Bedford, MA, USA) were blocked for 2 h with 5% non-fat milk. The membrane was then incubated with primary antibodies for 12 h. The blots were washed three times and incubated with secondary antibodies. After washing, the membranes were developed using an ECL Western blotting kit (Beyotime, Shanghai China). Finally, the blots were analysed quantitatively. The following antibodies were used: rabbit anti-Spry1 (1:1000; Abcam, MA, USA), rabbit anti-MMP13 (1:1000; Abcam, MA, USA), rabbit anti-VEGF (1:1000; Abcam, MA, USA), rabbit anti-ACAN (1:500; Abcam, MA, USA), rabbit anti-ERK (1:1000; Cell Signaling Technology, USA), rabbit anti-phospho-ERK (1:1000; Cell Signaling Technology, USA), rabbit anti-β-actin (1:1000; Beyotime, China), and rabbit anti-IgG (1:5000; Beyotime, China).

### Measurement of miRNAs and mRNA expression

Total RNA was extracted from the tissues and MCCs using TRIzol Reagent (Invitrogen). For quantitative detection of miRNA, a TaqMan miRNA assay kit (Thermo Fisher, USA) was used. Purified miRNA was reverse transcribed using miRNA-specific stem-loop RT primers (Applied Biosystems, USA). Following the manufacturer’s instructions, reverse transcription–quantitative PCR (RT-qPCR) was performed in a 7500 Real-Time PCR system (Applied Biosystems, USA) using SYBR® Premix Ex Taq II Kit (TaKaRa, Japan). Gene expression was normalized to U6 small nuclear RNA expression. The relative gene expression was measured by using the comparative threshold cycle (2^−ΔΔCt^) method, and β-actin served as an internal control. The reaction mixtures were incubated at 95 °C for 10 min, followed by 40 cycles of 20 s at 95 °C and 60 s at 55 °C. The primers used are shown in Supplementary 4 (the primer sequence of IL-1 β was supplemented in Supplementary 4).

### Toluidine blue staining

After treatment according to the experimental design, MCCs were washed three times with DPBS before staining, fixed in 4% buffered paraformaldehyde for at least 20 min at room temperature and washed with DPBS. Cells were then stained in toluidine blue solution for 10 min at 37 °C and washed with DPBS for 3 min. The staining results were observed by microscopy and quantified.

### Cell immunofluorescence

MCCs were washed with DPBS three times and fixed with 4% paraformaldehyde for 15 min and then rinsed in DPBS three times, 5 min each time. For Spry1 and ACAN detection, the cells were incubated with rabbit anti-Spry1 (1:100; Abcam, MA, USA) and rabbit anti-ACAN (1:50; Abcam, MA, USA) at 4 °C, overnight. FITC-labelled goat rabbit anti-IgG (Beyotime, Shanghai, China) was used as a secondary antibody and incubated for 1 h at 37 °C. The chondrocyte nuclei were restained with 4′, 6-diamidino-2-phenylindole (DAPI) (Solarbio, Beijing, China) for 10 min. The analysis of fluorescence was performed with a confocal scanning microscope system (Carl Zeiss, Nikon).

### Chick embryo in vivo angiogenesis assay

SPF-grade white eggs were purchased from Merial-Vital Company (Beijing, China). For the vascularization experiment, the cells were divided into four groups: NC, miR-21-5p inhibitor, miR-21-5p mimic, and miR-21-5p mimic + U0126. After 2 days of transfection, the cells of each group were resuspended in serum-free medium (15 μl) and mixed with BD Matrigel™ Basement Membrane Matrix (Corning, USA) of equal volume (15 μl each), and the mix was implanted into each egg, after which the eggs were returned to the incubator for further incubation. The embryos were incubated at 37.5 ± 0.5 °C and relative humidity of 60–80%. The position of the air chamber was marked on the surface of normal eggshells, part of the eggshell was peeled off to expose the egg white membrane and the intima was carefully removed with sterile tweezers. At embryonic day 10, Matrigel mixed with different groups of cells was implanted into each egg. After 72 h, images of the region surrounding the Matrigel were taken, and vascular branches were calculated.

### Statistical analysis

Bars represent the standard error of the mean (SEM) from three independent experiments. SPSS 20.0 and Image-Pro Plus 6.0 software were used for statistical and image analyses. The data were graphically presented using GraphPad Prism 7.0. Statistical significance between two groups was assessed by Student’s *t* test. For multiple comparisons, one-way analysis of variance (ANOVA) followed by Newman-Keuls post hoc tests was used. Data with a *p* value less than or equal to 0.05 were considered significant.

## Result

### Knockout of miR-21-5p reduces dentally induced TMJOA progression

**Fig. 1:**
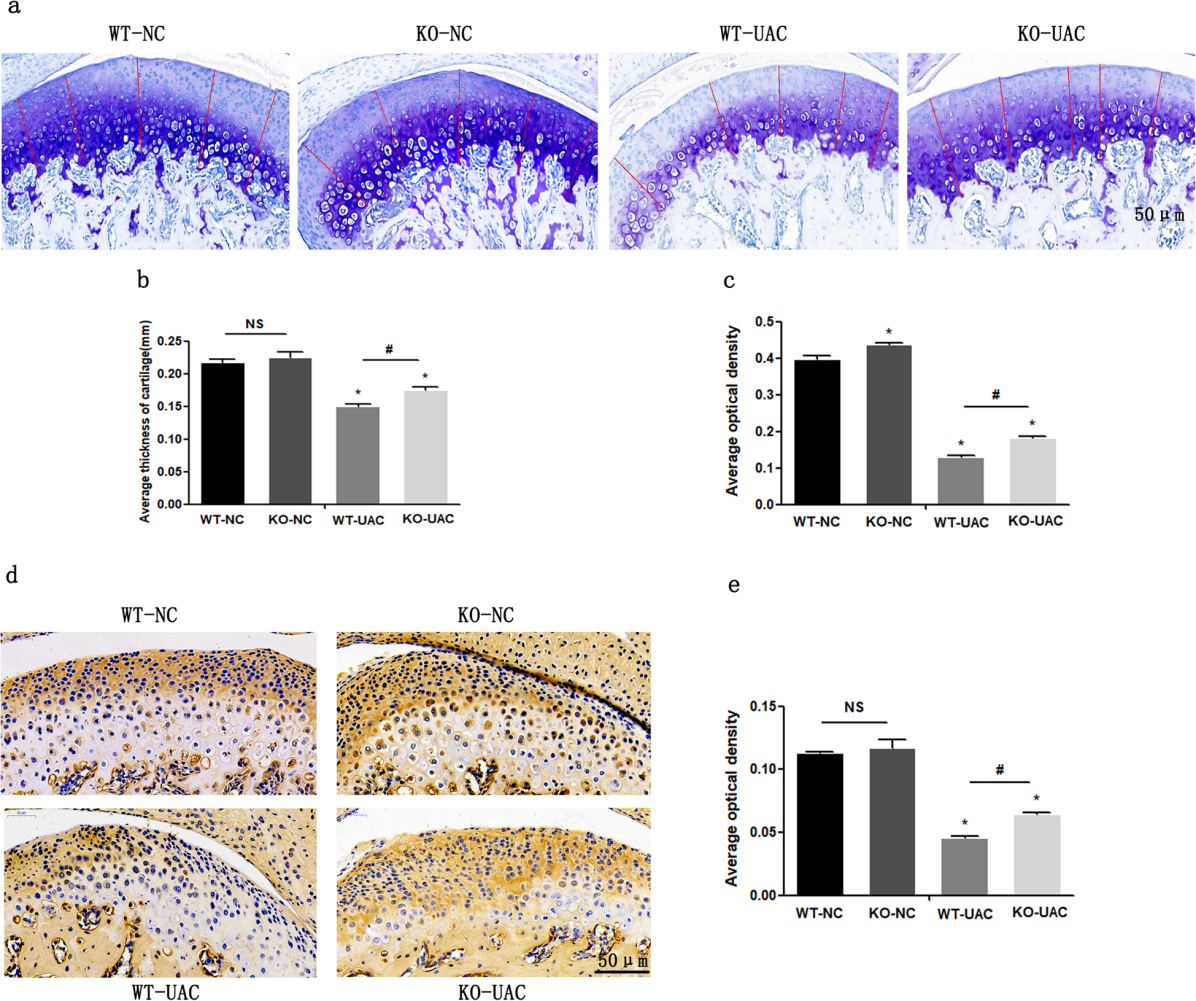
Toluidine blue (TB) and immunohistochemical (IHC) staining images of each section are shown. **a**–**d** Results showing that severe cartilage degeneration and loss of aggrecan (ACAN) of cartilage occurred in the unilateral anterior crossbite (UAC) group compared with the NC control group. In the UAC groups, the knockout (KO) UAC group had a reversal effect on cartilage degradation and ACAN loss. Comparison of the average optical density (AOD) (**b**) and the thickness of cartilage (**c**) of TB staining between groups. **e** Comparison of the AOD of IHC staining for ACAN between groups. Asterisk (*): compared with the wild-type negative control (WT-NC) group. Hash (^**#**^): compared with the wild-type UAC (WT-UAC) group. Data are represented as the means ± standard deviation (*n* = 3). *P < 0.05, ^**#**^*P* < 0.05. NS, not significant. Scale bar, 50 μm

Statistical analysis of Image-Pro Plus 6.0 software results showed that the AOD in the KO-UAC group was higher than that in the WT-UAC group (Fig. 1a, c). Furthermore, the results of the thicknesses of cartilage layers showed thinner layers in the WT-UAC group than in the KO-UAC group (Fig. 1a, b). There was no statistically significant difference between the WT-NC group and the KO-NC group in the AOD values and thickness of cartilage layers (Fig. 1a, b). In addition, we detected ACAN protein by immunohistochemistry (IHC) and determined that ACAN expression was significantly lower in the UAC group than in the NC group, and there was no significant difference between the WT-NC group and the KO-NC group; however, the expression of ACAN was higher in the KO-UAC group than in the WT-UAC group (Fig. 1d, e). These data suggest a chondroprotective effect of miR-21-5p KO on osteoarthritis mice and inhibition of cartilage matrix loss in UAC-induced OA.

### Knockout of miR-21-5p and UAC affect the expression of TMJ inflammatory-related molecules

**Fig. 2:**
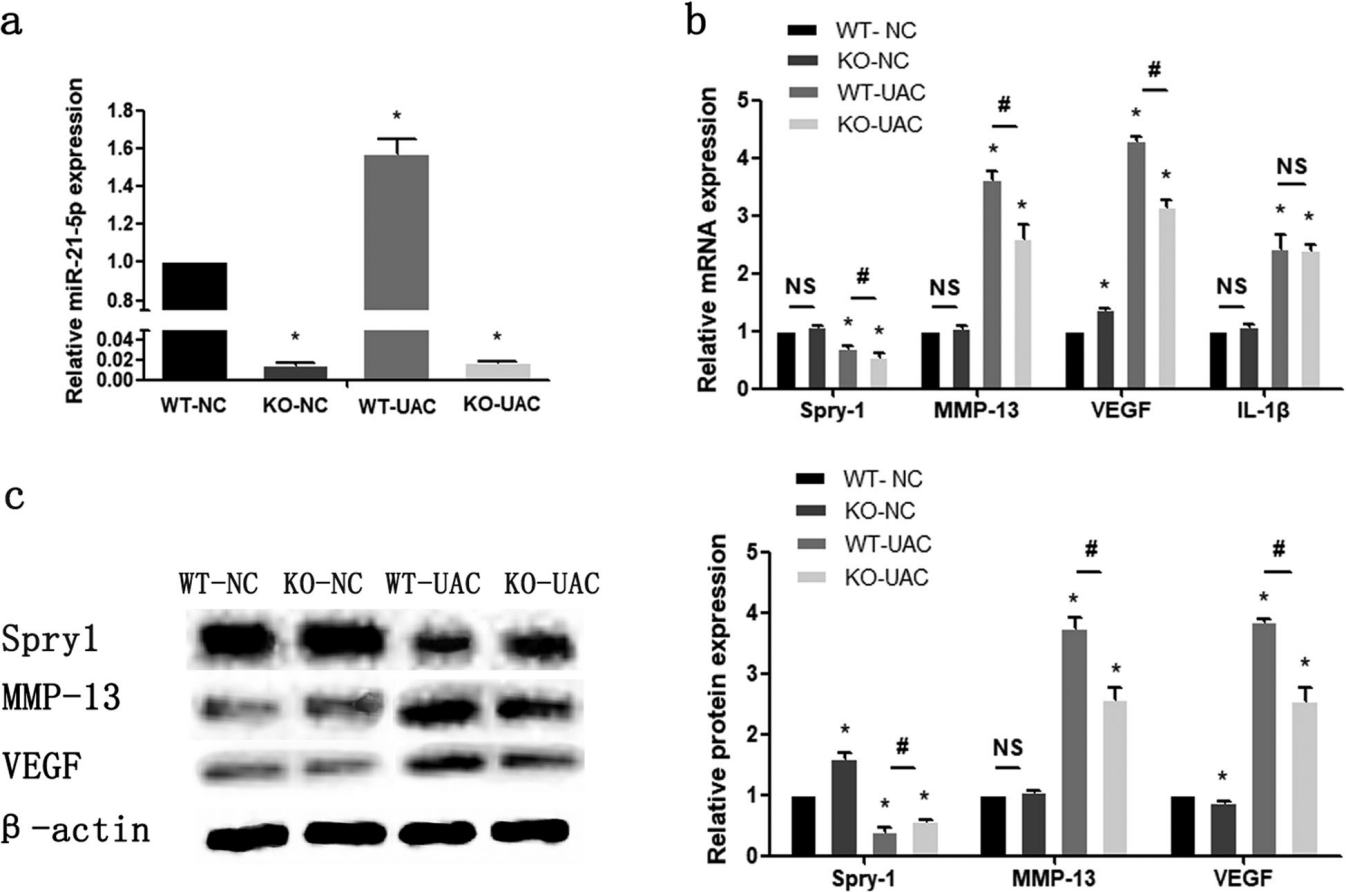
**a**, **b** Assessment of miR-21-5p, Spry1, MMP-13, VEGF, and IL-1β mRNA relative expression in cartilage of TMJ by qRT-PCR. Western blotting (**c**) results of Spry1, MMP-13 and VEGF protein expression in cartilage. Asterisk (*): compared with the WT-NC group. Hash (^**#**^): compared with the WT-UAC group. Data are represented as the means ± standard deviation (*n* = 3). *P < 0.05, ^**#**^*P* < 0.05. NS, not significant

As shown in Fig. 2, determined by qRT-PCR, the expression level of miR-21-5p was significantly decreased in the KO-NC and KO-UAC groups. MiR-21-5p, MMP-13, VEGF and IL-1β were increased in the WT-UAC group compared with the WT-NC group (Fig. 2a, b). Western blot results showed that the expression level of MMP-13 and VEGF were significantly increased in the WT-UAC when compared with the WT-NC and KO-NC groups, while compared with the WT-UAC group, knockout of miR-21-5p in the UAC group reduced MMP-13 and VEGF protein levels (Fig. 2c). Knockout of miR-21-5p increased Spry1 expression, while UAC treatment in a converse manner (Fig. 2c). These results suggest that dental induction of UAC promotes the expression of TMJOA-related molecules. In addition, knockout of miR-21-5p was associated with decreased expression of MMP-13 and VEGF and increased expression of Spry1 in UAC-induced OA.

### Spry1 was a target gene of miR-21-5p

**Fig. 3:**
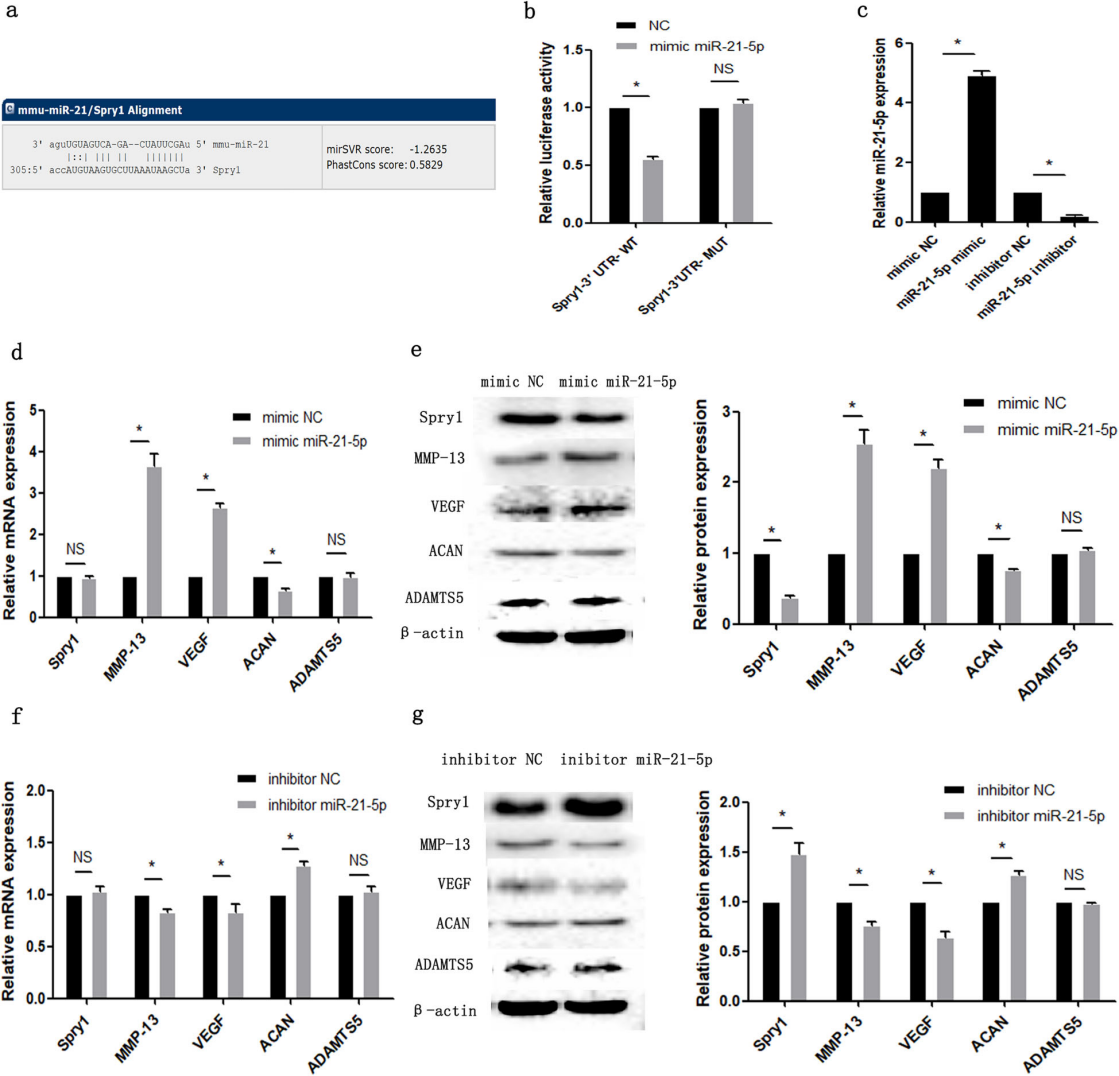
MiR-21-5p directly regulates the expression of Spry1 and affects the expression of specific genes in mandibular condylar chondrocytes (MCCs). **a** The predicted binding sites of miR-21-5p to the 3′ UTR of Spry1. **b** Dual-luciferase reporter assay showing that Spry1 is the target gene of miR-21-5p. Asterisk (*): compared with the negative control (NC) group. **c** Evaluation of the relative microRNA expression of miR-21-5p in MCCs transfected with mimic or inhibitor. RT-qPCR (**d**) and Western blotting (**e**) results of cartilage-specific gene expression in MCCs transfected with miR-21-5p mimic. RT-qPCR (**f**) and Western blotting (**g**) results of cartilage-specific gene expression in MCCs transfected with miR-21-5p inhibitor. Asterisk (*): compared with the negative control (NC) group. Data are represented as the means ± standard deviation (*n* = 3). *P < 0.05. NS, not significant

We first predicted that Spry1 was a target gene of miR-21-5p through analysis of miRNA target databases and identified the potential binding sequence of the 3′ UTR of Spry1 and miR-21-5p (Fig. 3a). To further confirm this result, we performed a dual-luciferase reporter assay. MCCs were transfected with miR-21-5p mimic and the corresponding negative control (NC) and then co-transfected with Spry1-3′ UTR-WT or Spry1-3′ UTR-MUT. Luciferase experiments showed that the Spry1-3′ UTR-WT group had a significant decrease in luciferase activity, but the Spry1-3′ UTR-MUT group had no significant change in luciferase activit**y** (Fig. 3b). This confirmed that Spry1 is a downstream target of miR-21-5p in chondrocytes. In addition, we found that Spry1 protein expression was significantly downregulated after transfection of MCCs with miR-21-5p mimic, while Spry1 protein expression was significantly upregulated after transfection of miR-21-5p inhibitor (Fig. 3e, g). These results support the hypothesis that Spry1 is a down-streamer of miR-21 in chondrocytes.

### The effect of miR-21-5p overexpression or silencing on the chondrocyte phenotype

MiR-21-5p mimic and inhibitor were transfected into MCCs to verify the role of miR-21-5p in MCCs and to detect changes in the mRNA and protein expression of Spry1, VEGF, MMP-13, ACAN and ADAMTS5. The RT-qPCR results showed that the expression of miR-21-5p was significantly increased in MCCs transfected with miR-21-5p mimic compared with the mimic NC group, while miR-21-5p was significantly inhibited in the miR-21-5p inhibitor group (Fig. 3c). The mRNA and protein expression levels of Spry1, VEGF and MMP-13 were significantly increased in the miR-21-5p mimic group compared with the mimic NC group, while the expression level of ACAN was downregulated (Fig. 3d, e). In contrast, the mRNA and protein expression of Spry1, VEGF and MMP-13 were decreased, and the expression of ACAN was upregulated in the miR-21-5p inhibitor group compared with the inhibitor NC group (Fig. 3f, g).

### IL-1β-induced degradation of the extracellular matrix of condylar cartilage is regulated by miR-21-5p

**Fig. 4:**
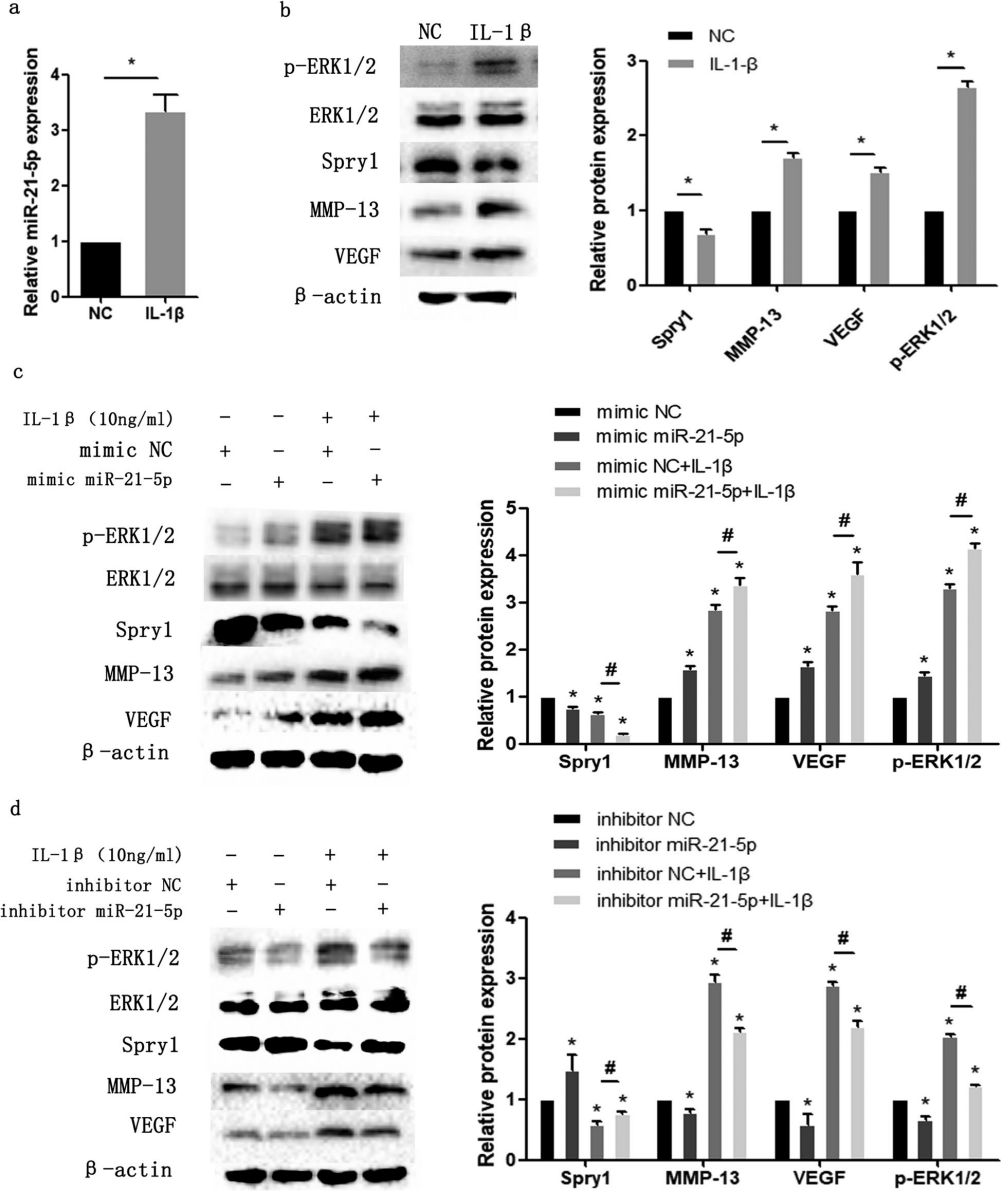
MiR-21-5p mediates the expression of related genes in IL-1β-induced condylar chondrocytes. **a** RT-qPCR detected the expression of miR-21-5p. **b** Protein expression levels of Spry1, MMP-13, VEGF and p-ERK1/2 were assayed by Western blot in MCCs under stimulation with IL-1β for 24 h. Western blotting results of Spry1, MMP-13, VEGF and p-ERK1/2 proteins in MCCs transfected with miR-21-5p mimic (**c**) or miR-21-5p inhibitor (**d**), followed by stimulation with IL-1β for 24 h. Asterisk (*): compared with mimic NC (**b**, **d**) or inhibitor NC group (**c**, **e**). Hash (^**#**^): compared with the mimic NC + IL-1β or inhibitor NC + IL-1β group. Data are represented as the means ± standard deviation (*n* = 3). *P < 0.05, ^**#**^*P* < 0.05. NS, not significant

**Fig. 5:**
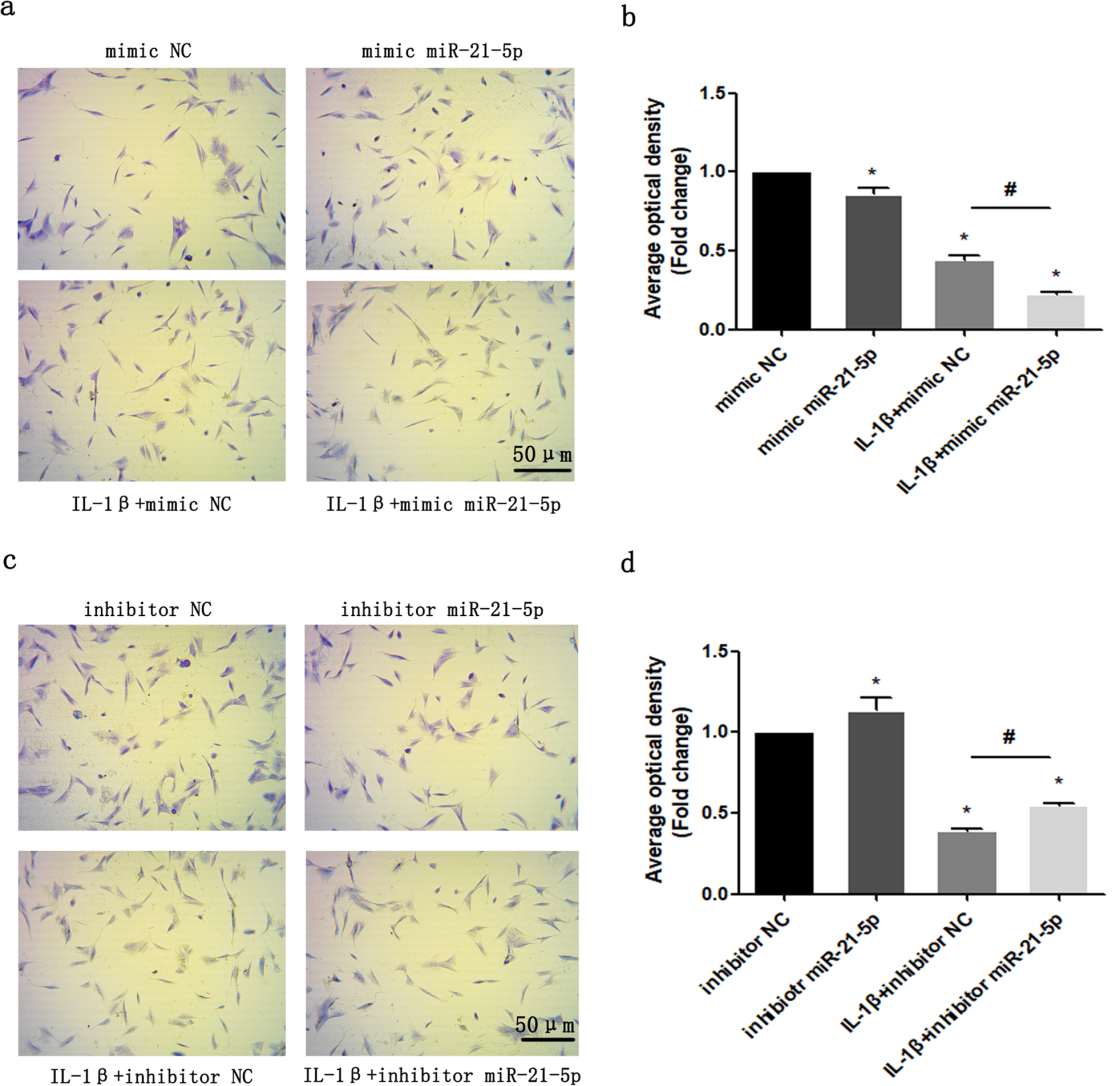
Toluidine blue staining results of MCCs transfected with miR-21-5p mimic (**a**) or miR-21-5p inhibitor (**c**), followed by stimulation with IL-1β for 24 h. Scale bar, 50 μm (magnification, × 200). Lower panel, statistical analysis of the average optical density of matrix staining by toluidine blue. Asterisk (*): compared with the mimic NC (**b**) or inhibitor NC group (**d**). Hash (^**#**^): compared with the mimic NC + IL-1β or inhibitor NC + IL-1β group. Data are represented as the means ± standard deviation (*n* = 3). *P < 0.05, ^**#**^*P* < 0.05. NS, not significant. Scale bar, 50 μm

IL-1β was used to induce an inflammatory state in MCCs. As shown in Fig. 4, the expression level of miR-21-5p was significantly increased in IL-1β-treated MCCs (Fig. 4a). The protein expression of the corresponding target gene Spry1 was inhibited, and the expression levels of VEGF, MMP-13 and p-ERK1/2 were significantly increased (Fig. 4b). To demonstrate that miR-21-5p can promote the degradation of extracellular matrix under the inflammatory induction of IL-1β, we transfected MCCs with miR-21-5p mimic or miR-21-5p inhibitor, and the corresponding group was treated with IL-1β for 24 h after transfection. Under treatment with IL-1β, the expression of VEGF, MMP-13 and p-ERK1/2 in the miR-21-5p mimic group was significantly upregulated (Fig. 4c), while the upregulation of VEGF, MMP-13 and p-ERK1/2 was significantly attenuated in the miR-21-5p inhibitor group (Fig. 4d). In addition, we evaluated the change in matrix content by performing toluidine blue staining in MCCs. The results showed that the average optical density values of the IL-1β-treated group were lower than those of the untreated group. Compared with the negative control, overexpression of miR-21-5p reduced matrix staining, while silencing miR-21-5p increased matrix staining, especially in the presence of IL-1β-induced MCCs (Fig. 5a–d). These results suggest that miR-21-5p promotes IL-1β-mediated extracellular matrix catabolism.

### IL-1β induces degradation of the extracellular matrix of MCCs and can be regulated by miR-21-5p-targeting Spry1

**Fig. 6:**
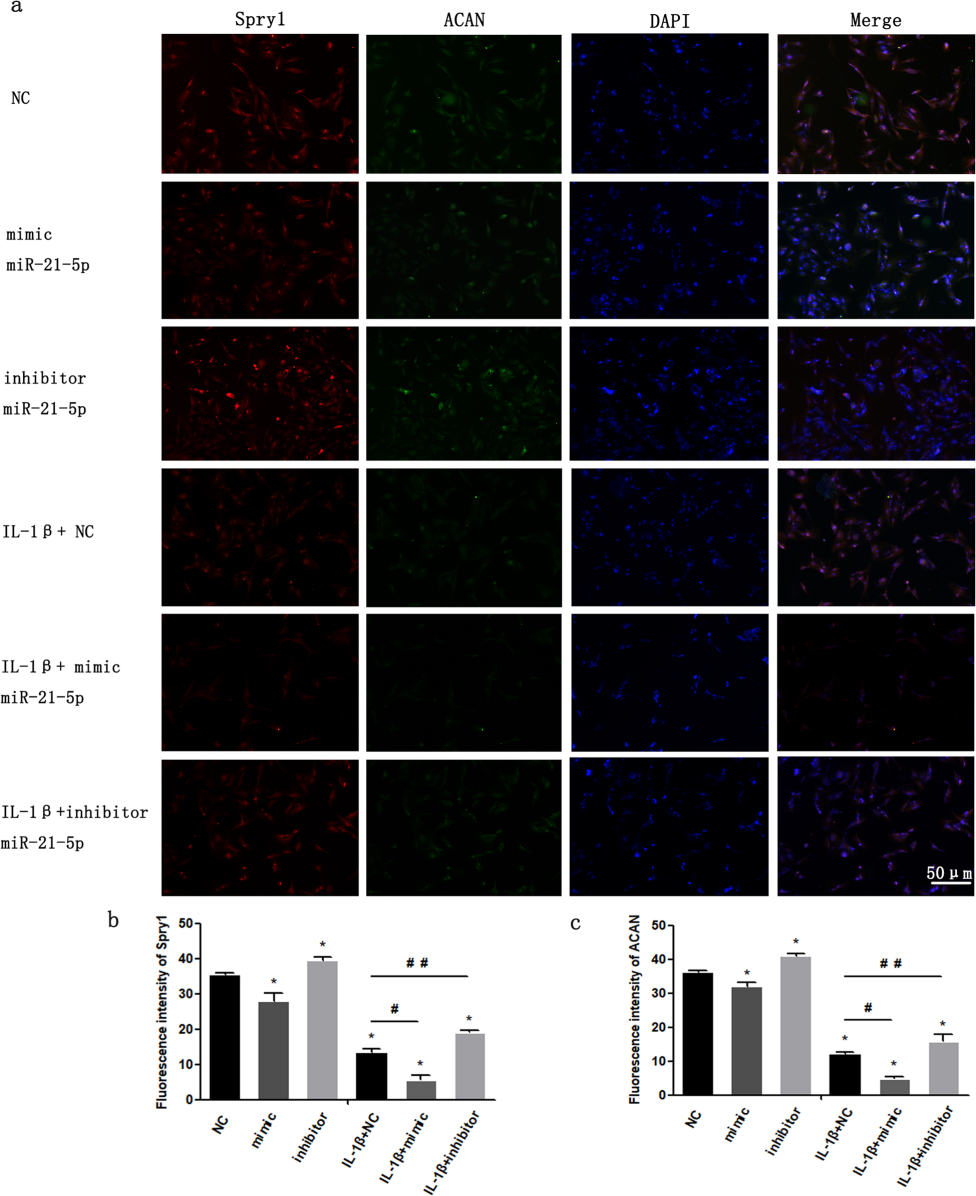
**a** Immunofluorescence intensity analysis of Spry1 and ACAN expression in MCCs. MCCs were transfected with miR-21-5p mimic or miR-21-5p inhibitor and then stimulated with IL-1β for 24 h. Fluorescence micrographs of Spry1 are shown in red (**b**), ACAN (**c**) in green, and nuclei in blue. Data are represented as the means ± standard deviation (*n* = 3). Asterisk (*): compared with the NC group. Hash (^**##**^): compared with the IL-1β + NC group. Data are represented as the means ± standard deviation (*n* = 3). *P < 0.05, ^**#**^*P* < 0.05, ^**##**^*P* < 0.05. NS, not significant. Scale bar, 50 μm

We transfected MCCs with miR-21-5p mimic or inhibitor and then treated the cells with IL-1β according to the experimental groups. After that, Spry1 and ACAN were stained by immunofluorescence (Fig. 6). Compared with that in the NC group, the fluorescence intensity of Spry1 and ACAN in the miR-21-5p mimic group, IL-1β + NC group and IL-1β + mimic group was significantly decreased. In addition, the fluorescence intensity of Spry1 and ACAN in the IL-1β + inhibitor group was significantly higher than that in the IL-1β + NC group and the IL-1β + mimic group. More importantly, the fluorescence intensity of Spry1 and ACAN in the IL-1β + mimic group was significantly lower than that in the miR-21-5p mimic group and the IL-1β + NC group. Therefore, we believe that the process by which miR-21-5p promotes the degradation of the MCC extracellular matrix is likely to be related to its targeted regulation of Spry1 expression.

**Fig. 7:**
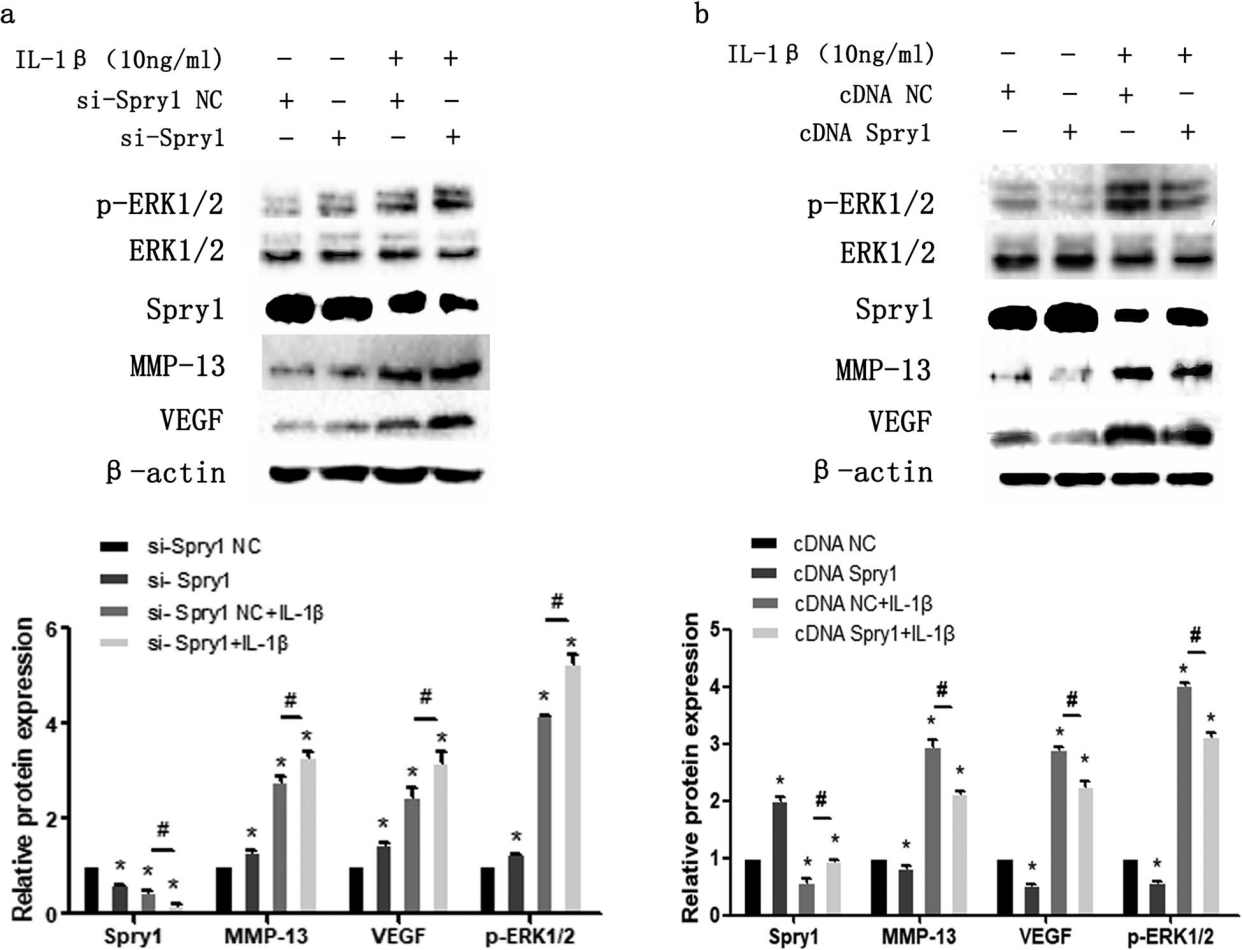
MiR-21-5p could affect the expression of MCC inflammatory factors by regulating Spry1. **a** MCCs transfected with Spry1 siRNA (si-Spry1) or negative control (si-NC) were stimulated by 10 ng/ml IL-1β for 24 h. **b** MCCs transfected with empty vector (cDNA NC) or Spry1 ectopic expression plasmid (cDNA Spry1) for 24 h were stimulated by IL-1β for another 24 h. Asterisk (*): compared with the si-NC (**a**) or cDNA NC (**b**) group. Hash (^**#**^): compared with the si-NC + IL-1β (**a**) group or cDNA NC + IL-1β (**b**) group. Western blot analysis showing the expression of Spry1, MMP-13, VEGF and p-ERK1/2 in MCCs (**a**, **b**). Data are represented as the means ± standard deviation (*n* = 3). *P < 0.05, ^**#**^*P* < 0.05. NS, not significant

**Fig. 8:**
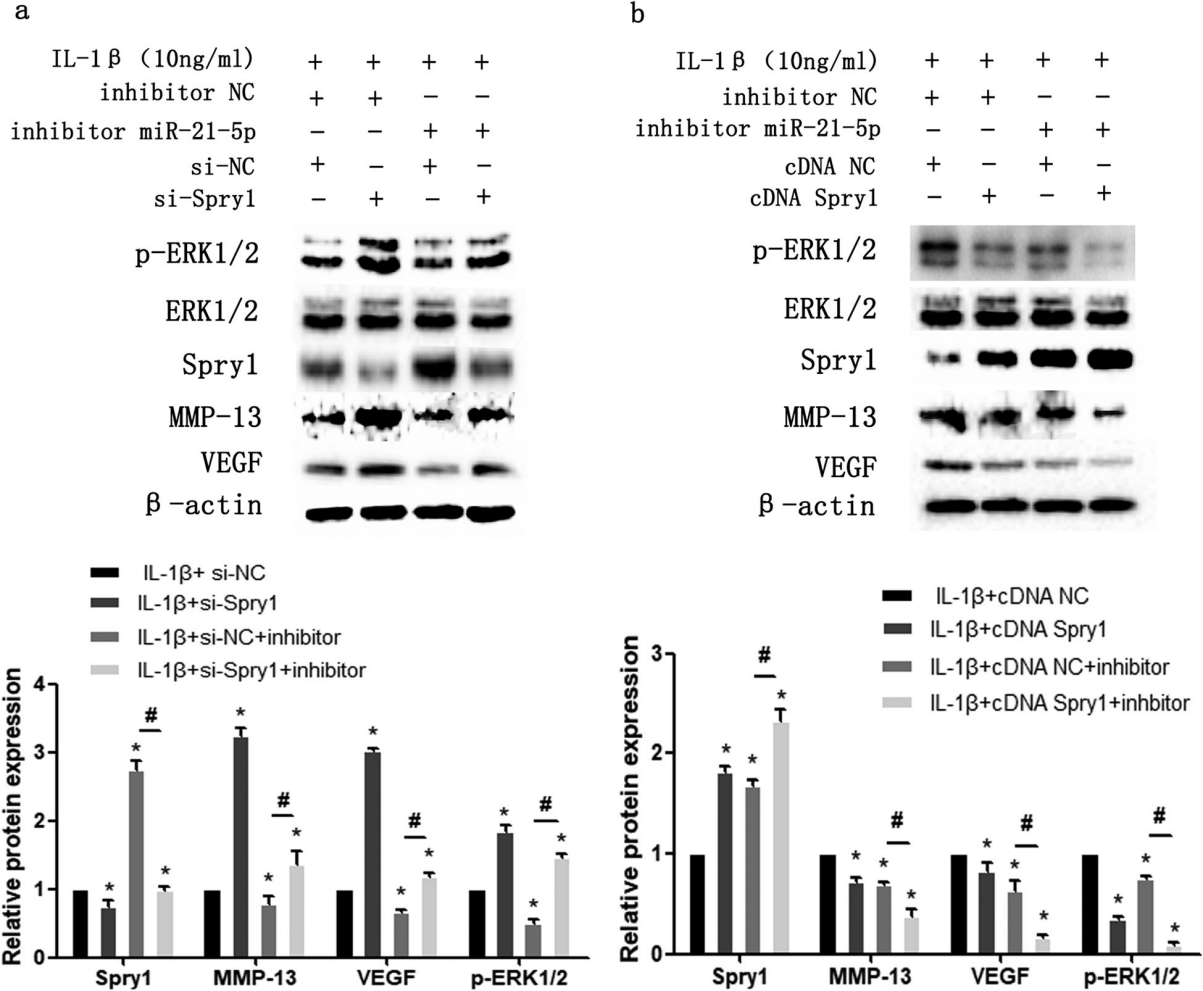
**a** Under stimulation with IL-1β, MCCs were transfected with inhibitor NC or miR-21-5p inhibitor for 24 h and then treated with Spry1 siRNA (si-Spry1) for another 24 h. **b** Under stimulation with IL-1β, MCCs were treated with inhibitor NC or miR-21-5p inhibitor for 24 h and then transfected with the empty vector (cDNA NC) or Spry1 ectopic expression plasmid (cDNA Spry1) for another 24 h. Asterisk (*): compared with the IL-1β + si-NC group (**a**) or IL-1β + cDNA NC group (**b**). Hash (^**#**^): compared with the IL-1β + si-NC + inhibitor group (**a**) or IL-1β + cDNA NC + inhibitor group (**b**). Western blot analysis showing the expression of Spry1, MMP-13, VEGF and p-ERK1/2 in MCCs (**a**, **b**). Data are represented as the means ± standard deviation (*n* = 3). *P < 0.05, ^**#**^*P* < 0.05. NS, not significant

To further demonstrate that miR-21-5p promotes extracellular matrix degradation under an inflammatory state through regulation of its target gene Spry1, we used small interfering RNA (si-Spry1) and a Spry1 ectopic expression plasmid (cDNA Spry1) to transfect MCCs. The knockdown of Spry1 expression induced by IL-1β significantly increased the expression of VEGF and MMP-13 and promoted the activation of the ERK/MAPK signalling pathway (Fig. 7a). Transfection of ectopic expression plasmids showed that the expression of VEGF, MMP-13 and p-ERK1/2 in the IL-1β + cDNA Spry1 group was significantly downregulated compared with that in the IL-1β + cDNA NC group (Fig. 7b). In addition, we also co-transfected miR-21-5p inhibitor with Spry1 small interfering RNA and ectopic expression plasmid and showed that overexpression of Spry1 could enhance the inhibitory effect of miR-21-5p inhibitor on VEGF, MMP-13 and p-ERK1/2 protein expression levels in the inflammatory state (Fig. 8b). Interfering with the expression of Spry1 attenuated the inhibition (Fig. 8a). This indicates that miR-21-5p affects the degradation of extracellular matrix induced by IL-1β via the targeted regulation of Spry1.

### MiR-21-5p promotes angiogenesis in MCCs that is mediated by the ERK/MAPK signalling pathway

**Fig. 9:**
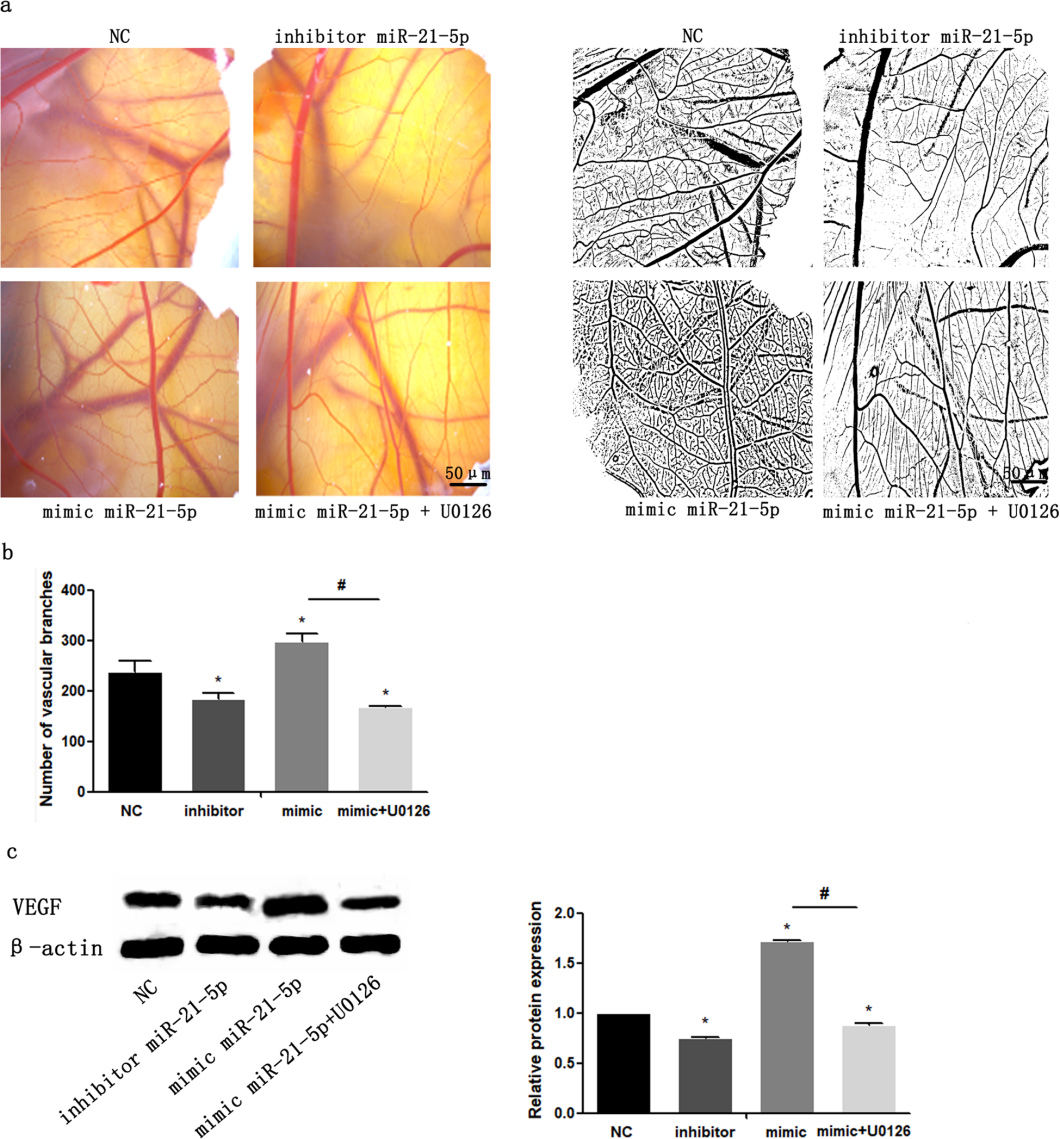
MiR-21-5p enhances the angiogenesis potential of MCCs by activating the ERK/MAPK signalling pathway. **a**, **c** MCCs were transfected with miR-21-5p mimic or miR-21-5p inhibitor and treated with U0126. **a** CAM representative images showing the inoculation area of the different experimental conditions. **b** Quantitative evaluation of the number of new blood vessels grown towards each inoculation area. Detection of VEGF protein expression changes (**c**). Data are represented as the means ± standard deviation (*n* = 8) (**b**). Data are represented as the means ± standard deviation (*n* = 3) (**c**). Asterisk (*): compared with the negative control (NC) group. Hash (^**#**^): compared with the mimic group. *P < 0.05, ^**#**^*P* < 0.05. NS, not significant. Scale bar, 50 μm

To detect the effect of miR-21-5p on the angiogenesis of TMJ cartilage and to verify whether the ERK-MAPK signalling pathway is involved in the process of miR-21-5p-induced angiogenesis, we transfected MCCs with miR-21-5p mimic or inhibitor, and the ERK-MAPK inhibitor U0126 was used in MCCs transfected with miR-21-5p mimic. The number of blood vessels in each group of eight chicken embryos was measured. Compared with that in the NC group, the number of vascular branches in the miR-21-5p mimic group increased significantly, while that in the miR-21-5p inhibitor group was the smallest. In addition, the number of new vessels in the miR-21-5p mimic and U0126 co-intervention group was significantly lower than that in the miR-21-5p mimic group but was closer to that in the NC group (Fig. 9a, b) (Table 2). To verify that U0126 can significantly inhibit the activation of p-ERK1/2, the expression of p-ERK protein was detected (Supplementary 5). Then, the expression of VEGF in the mimic + U0126 group was significantly lower than that in the mimic group (Fig. 9c), which indicated that the activation of the ERK-MAPK signalling pathway in MCC is likely to regulate angiogenesis by promoting VEGF expression. Based on the above results, we believe that the expression of miR-21-5p in MCCs promotes the angiogenic activity of endothelial cells, which is at least partially mediated by the ERK-MAPK signalling pathway.

**Table 2:** Number of vascular branches in different groups

Embryo number^#^	NC	Inhibitor	mimic	mimic + U0126
1^#^	159	217	289	138
2^#^	220	182	270	187
3^#^	244	197	291	178
4^#^	249	212	290	210
5^#^	269	207	260	168
6^#^	225	112	316	137
7^#^	286	127	287	148
8^#^	239	162	299	198
Mean ± standard deviation	236.38 ± 38.05	188.25 ± 36.23	287.75 ± 15.9	170.50 ± 27.61

*Inhibitor* microRNA-21-5p inhibitor, *mimic* microRNA-21-5p mimic,* #* The number of each chicken embryo

## Discussion

At present, researches on the mechanism of microRNA regulation of OA disease development mainly focuses on large joints, such as knee, hip and finger joints, and there is little research on the mechanism of TMJOA. TMJ belongs to a kind of special linkage joint, which is different from other joints in anatomy and tissue structure, so TMJ also has potential research value. In addition, we found Spry1 mediated in the regulation of TMJOA by miR-21-5p. The innovation of this experiment is to reveal the potential role of miR-21-5p and its target gene Spry1 in the progression of TMJOA disease and to provide the possibility for the development of TMJOA treatment strategies in the future. Our experiments aimed to investigate the possible mechanism of action of miR-21-5p in condylar cartilage inflammatory degeneration by establishing a UAC mouse model to induce cartilage structural dysfunction and using IL-1β to induce the inflammatory state of MCCs.

In recent years, the UAC model has been widely used to induce TMJOA, that is, to change the occlusal load distribution and induce OA through experimental unilateral anterior cross occlusion, leading to the loss of subchondral bone in the TMJ [20–22]. In vivo experiments confirmed that UAC induced TMJ cartilage degeneration and found that MMP-13, VEGF and IL-1β were upregulated. In addition, knockout of miR-21-5p has been shown to have significant attenuation effects on cartilage degradation. It provides basic evidence for the following experiments. In vitro experiments have used the inflammation-inducing factor IL-1β, which is closely related to bone and cartilage loss in OA, and many studies have demonstrated that the MMP induction by IL-1β is mediated by the ERK-MAPK signalling pathway [23–25]. ERK1/2 is involved in a variety of cellular responses in the bone and cartilage and is one of the important members of the MAPK cascade. It is well known that VEGF, a classical angiogenic factor, is closely related to the progression of OA disease. It has been confirmed that when VEGF is combined with its signal receptor VEGF2, downstream kinases such as MAPK can be phosphorylated to activate the ERK-MAPK signalling pathway [26]. Moreover, ERK-MAPK signalling pathway activated by VEGF can induce the activation of MMPs [27]. Because VEGF, MMPs and the ERK-MAPK pathways are closely related to the development of OA and there is a close relationship between them, they are regarded as the OA phenotypes in our vitro experiments.

MicroRNAs determine cell fate by participating in the regulation of extracellular signalling pathways and expression of certain molecules [28], and miR-21-5p has been reported to play an important role in cell apoptosis, pathological growth and stress [29, 30], processes that are closely related to OA cartilage lesions. Some researchers have found that miR-21-5p plays an important role in the development of inflammation[31–33], and there have been many studies on the involvement of miR-21-5p in the progression of various diseases through its target genes Spry1 and Spry2 [15, 28, 34]. More importantly, Wang et al. [13] and Zhang et al. [14] have recently demonstrated that miR-21-5p promotes the progression of OA by targeting growth differentiation factor 5 (GDF5) and FGF18, respectively. At present, there have been no studies on the regulatory mechanism of miR-21-5p and its target gene Spry1 in OA. However, many research groups have confirmed that Spry1 can inhibit the phosphorylation of the ERK-MAPK signalling pathway and the expression of VEGF [15, 16, 35–38]. Since miR-21-5p, ERK-MAPK and VEGF are closely related to OA, we hypothesized that Spry1 is likely to act as a link between miR-21-5p, ERK-MAPK and VEGF and to play a regulatory role in the development of TMJOA. We established a UAC model that can induce TMJOA in vivo. The staining results suggest that KO miR-21-5p can delay or even inhibit the development of TMJOA disease induced by surgery. The dual-luciferase assay results showed that miR-21-5p could also directly target the expression of Spry1 in cartilage. Then, by transfecting MCCs with miR-21-5p mimic, we demonstrated that upregulation of miR-21-5p inhibited the expression of Spry1 protein and promoted the secretion of VEGF and MMP-13 and the phosphorylation of ERK-MAPK pathway components. Conversely, inhibition of miR-21-5p had the opposite effects. We induced the inflammatory state of MCCs by IL-1β and found that miR-21-5p inhibitor could attenuate the effect of IL-1β on the expression of VEGF, MMP-13 and p-ERK1/2 and upregulate the expression of the target gene Spry1 of miR-21-5p, while overexpression of miR-21-5p had the opposite effect. It is suggested that miR-21-5p may be involved in the catabolism of MCCs by targeting Spry1. The positive correlation between miR-21-5p and OA is also consistent with the conclusions of Wang et al. [13] and Zhang et al. [14]. To further clarify that miR-21-5p promotes the secretion of VEGF and MMP-13 and phosphorylation of ERK-MAPK signalling pathway components by targeting Spry1, we co-transfected MCCs with si-Spry1 and miR-21-5p inhibitor or with cDNA-Spry1 and miR-21-5p inhibitor. Interestingly, overexpression of Spry1 enhanced the protective effect of miR-21-5p inhibitor on TMJOA. These results indicate that miR-21-5p promotes the degradation of the extracellular matrix by targeting Spry1 to upregulate the expression of VEGF and MMP-13 and phosphorylation of ERK-MAPK signalling pathway components in inflammatory MCCs. It is worth noting that there is a significant positive correlation between VEGF and miR-21-5p in vitro. Moreover, according to many studies, miR-21-5p can regulate the phosphorylation of ERK-MAPK signalling pathway components by targeting Spry1 in a variety of cells [15, 34, 38, 39] and based on the close correlation between VEGF and the ERK-MAPK signalling pathway [7, 27, 38], we speculate that miR-21-5p can promote angiogenesis in TMJ, and this effect is likely related to the degree of phosphorylation of ERK-MAPK signalling pathway components. Finally, the experimental results of the chicken embryo angiogenesis assay and Western blotting showed that miR-21-5p has a pro-angiogenic effect, which was significantly mediated by the ERK-MAPK signalling pathway.

This experiment only verified that miR-21-5p-targeting Spry1 can simultaneously increase the expression of VEGF, MMP-13 and p-ERK1/2. However, in this signalling axis, whether VEGF is upstream or downstream of ERK-MAPK and whether VEGF has a feedback-promoting or -inhibiting effect on the expression of Spry1 need further experimental verification. In addition, due to experimental constraints, we could not obtain enough human TMJ samples in a short time, which is a clinical limitation of our study.

## Conclusions

To the best of our knowledge, this is the first report of miR-21-5p affecting the progression of TMJOA disease in mice by targeting Spry1. We concluded that KO miR-21-5p attenuates the deterioration of condylar cartilage structure and function caused by the mouse UAC model. Furthermore, in the inflammatory state induced by IL-1β, miR-21-5p can promote the degradation of extracellular matrix and angiogenesis in TMJOA by downregulating the expression of Spry1. These findings not only provide new insights into the role of Spry1, a classical inhibitor of the Ras/Raf/ERK signalling pathway, in regulating TMJOA matrix degradation and angiogenesis but also provide a new strategy for the future use of miRNAs in TMJOA treatment.

## Supplementary information

**Additional file 1: Supplementary 1** Identification of knockout mice

**Additional file 2: Supplementary 2** Primary mouse condylar chondrocytes (MCCs) and immunocytochemical identification of type II collagen.

**Additional file 3: Supplementary 3** The target gene sequence for chemical synthesis and the primer sequence for identification of the recombinant plasmid.

**Additional file 4: Supplementary 4** Primer sequences of related genes for reverse transcription quantitative polymerase chain reaction.

**Additional file 5: Supplementary 5** To verify that U0126 can significantly inhibit the activation of p-ERK1/2, the expression of p-ERK protein was detected.

## Data Availability

The datasets used and/or analysed during the study are available from the corresponding author on reasonable request.

## Abbreviations

TMJOA: Temporomandibular joint osteoarthritis
TMJ: Temporomandibular joint
miRs: MicroRNAs
Spry1: Sprouty1
UAC: Unilateral anterior crossbite
CAM: Chorioallantoic membrane
MCCs: Mandibular condylar chondrocytes
ACAN: Aggrecan
VEGF: Vascular endothelial growth factor
MMPs: Matrix metalloproteinases
3′ UTR: 3′-Untranslated region
FGF18: Fibroblast growth factor 18
KO: Knockout
MiR-21-5p KO: MiR-21-5p^**−/−**^C57BL/6 knockout
MiR-21-5p: MicroRNA-21-5p
WT: Wild-type
NC: Negative control
IOD: Integrated optical density
AREA: The area of the pixel
BSA: Bovine serum albumin
mimic NC: Mimic negative control
inhibitor NC: Inhibitor negative control
si-Spry1: Small interference RNA of mouse Spry1
si-NC: Negative control siRNA
cDNA Spry1: Spry1 ectopic expression plasmid
Ct: Comparative threshold cycle
GADPH: Glyceraldehyde 3-phosphate dehydrogenase
DAPI: 4′, 6-Diamidino-2-phenylindole
SEM: Standard error of the mean
ANOVA: One-way analysis of variation
IHC: Immunohistochemistry
qRT-PCR: Real-time quantitative reverse transcriptase polymerase chain reaction
GDF5: Growth differentiation factor 5
