# Supplementary material for: MiR-21-5p regulates extracellular matrix degradation and angiogenesis in TMJOA by targeting Spry1
Source: Arthritis Res Ther. 2020 May 1;22:99. doi: 10.1186/s13075-020-2145-y (PMC7195789; doi:10.1186/s13075-020-2145-y)
Supplement: Supplementary file 1 — Additional file 1: Supplementary 1 Identification of knockout mice [file 13075_2020_2145_MOESM1_ESM.docx]

**Supplementary 1**

Identification of knockout mice

| primer | \| 1540 \| Mir21-tF2 \| CTTACTTCTCTGTGTGATTTCTGTG \| -94bp=451bp Wt=545bp \|  \| \| --- \| --- \| --- \| --- \| --- \| \| 1541 \| Mir21-tR2 \| ACAACCTTTCCAAAATCCATGAGGC \| | | |
| --- | --- | --- | --- | --- | --- | --- | --- | --- | --- | --- | --- |
| PCR  system | \| **Reaction Components** \| **Volume （μL）** \| \| --- \| --- \| \| gDNA Template \| 2.0 \| \| 10×*Taq* Buffer(mg^2+^ plus) \| 2.0 \| \| dNTP Mixture（10 mM） \| 0.5 \| \| Primer mix（10 μM） \| 0.5 \| \| *Taq* DNA polymerase(5 U/µL) \| 0.5 \| \| Milli-Q H_2_O \| To 20μL \| |  | |
| PCR process | \| **Seg.** \| **Temp.** \| **Time** \| **Cycle** \| \| --- \| --- \| --- \| --- \| \| 1 \| 95^o^C \| 5min \|  \| \| 2 \| 95 ^o^C \| 30s \|  \| \| 3 \| 58^o^C \| 30s \|  \| \| 4 \| 72 ^o^C \| 45s \| 2-4,40 \| \| 5 \| 72 ^o^C \| 3min \|  \| \| 6 \| 25 ^o^C \| hold \|  \|   **P1** | |  |
| Gel concentration | **1.5%** | | |
| Electrophoregram | **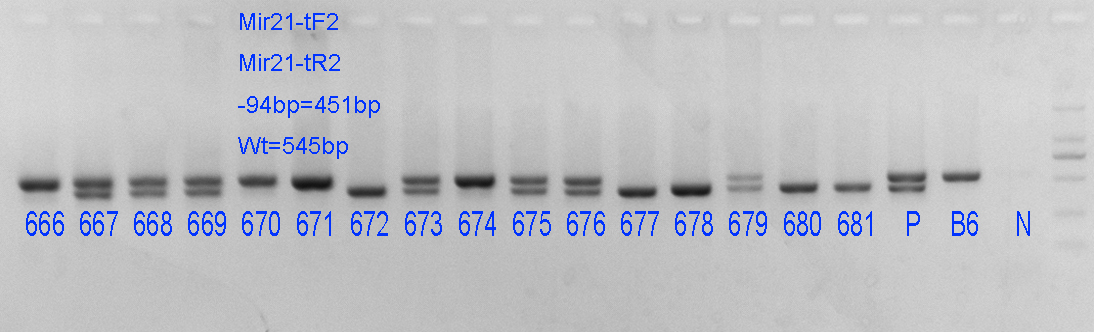** | | |
| Result judgement | **-94bp/-94bp：672，677，678，680，681**  **-94bp/wt：667, 668, 669，673，675，676，679**  **Wt/wt：666, 670, 671, 674** | | |
