# Supplementary material for: MiR-21-5p regulates extracellular matrix degradation and angiogenesis in TMJOA by targeting Spry1
Source: Arthritis Res Ther. 2020 May 1;22:99. doi: 10.1186/s13075-020-2145-y (PMC7195789; doi:10.1186/s13075-020-2145-y)

**Supplementary 2**

Primary mouse condylar chondrocytes (MCCs) and immunocytochemical identification of type II collagen.


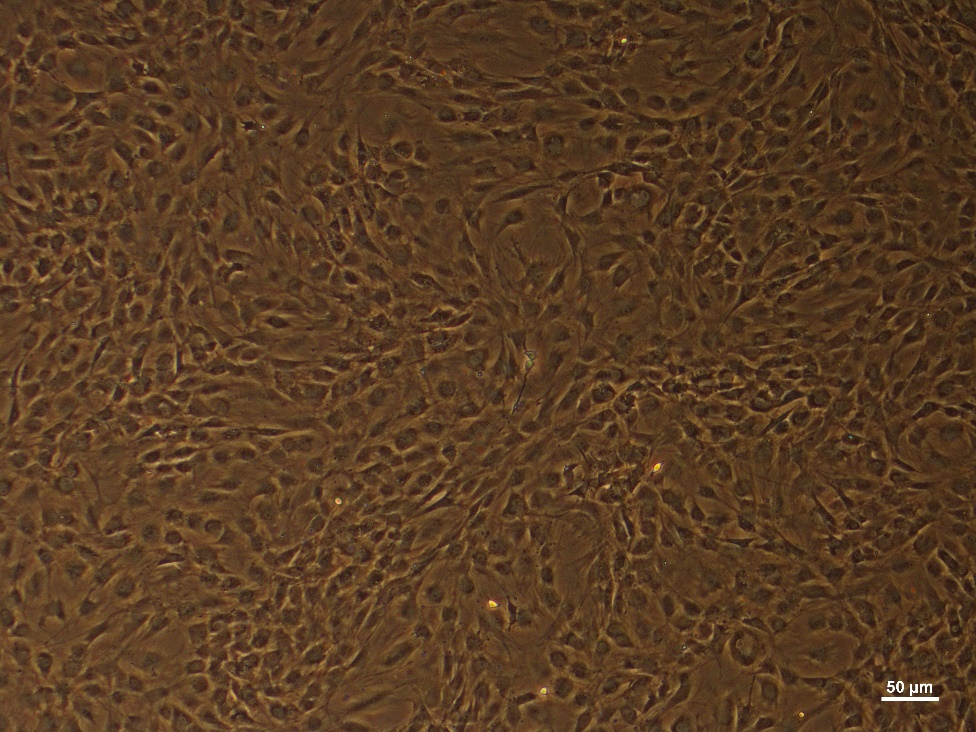


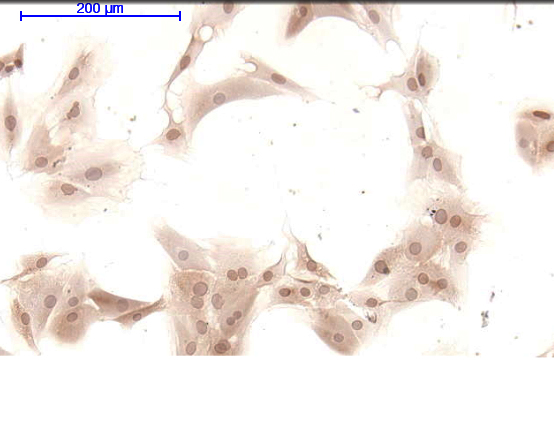

Supplement: Supplementary file 2 — Additional file 2: Supplementary 2 Primary mouse condylar chondrocytes (MCCs) and immunocytochemical identification of type II collagen. [file 13075_2020_2145_MOESM2_ESM.docx]
