# Supplementary material for: MiR-21-5p regulates extracellular matrix degradation and angiogenesis in TMJOA by targeting Spry1
Source: Arthritis Res Ther. 2020 May 1;22:99. doi: 10.1186/s13075-020-2145-y (PMC7195789; doi:10.1186/s13075-020-2145-y)
Supplement: Supplementary file 3 — Additional file 3: Supplementary 3 The target gene sequence for chemical synthesis and the primer sequence for identification of the recombinant plasmid. [file 13075_2020_2145_MOESM3_ESM.docx]

**Supplementary 3 The target gene sequence for chemical synthesis and the primer sequence for identification of the recombinant plasmid.**

The vector for Spry1：GV141

Cloning site：XhoI / KpnI

| **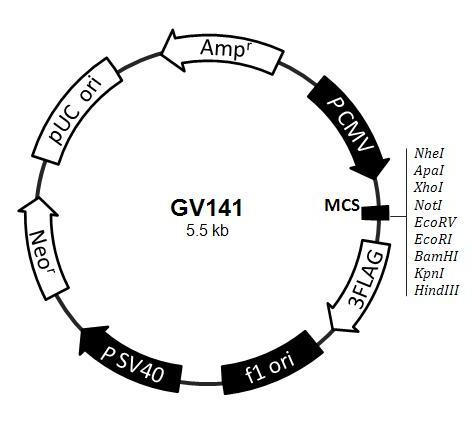** |
| --- |

The target gene sequence of chemical synthesis.

CTCGAGCGCCACCATGGATTCCCCAAGTCAGCATGACAGCCACACTTCACTAGTGGTGATTCAGCCACCGGCTGTGGAAGGCCGGCAGAGGTTAGACTATGACAGGGACACCCAGCCTGCCACGATTCTGTCCCTAGACCAGATCAAAGCCATCAGAGGCAGCAATGAATACACAGAGGGACCTTCGGTAGCGAGAAGACCAACACAGTTTTCTTGGCATCTACTCACTGCCTTGTCCTTACAGCCCTCCGCAATCTCCCCACAATGCACTGTGCAGATGGGTCATCCCCGCACACAGCTCTGGATTCACTGTGCGGTGTTGGTCTTCACATCAGAGATGATGTTGGCAACCCTCCTTTCTCCTCCTCCAGCACCCAGTATCCGTGAAGGCCTTCCCTTCGCACCCCCCACTTCACTGAGGGGGCGCTTGGACACGGTAGTTGACATTCCTATCTCGGTACC

The primer sequence for identification.

| **ID** | **seq** |
| --- | --- |
| CMV-F | CGCAAATGGGCGGTAGGCGTG |
| pcDNA-SEQR | TTATTAGGAAAGGACAGTGGG |

PCR identification of recombinant clone

**1 2 3 4 5 6 7 8 9 10 11 12**

| **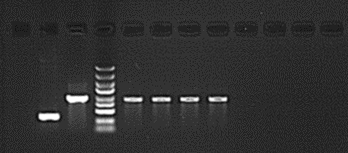** |
| --- |

电泳图说明：

1#：negative control（ddH2O）

2#：Negative control group (no load self connected control group)

3#：positive control（GAPDH）

4#：Marker: 5 kb，3 kb，2 kb，1.5 kb，1 Kb，750 bp，500 bp，250 bp，100 bp

5-12#:1-8 convertor.
