# Supplementary material for: MiR-21-5p regulates extracellular matrix degradation and angiogenesis in TMJOA by targeting Spry1
Source: Arthritis Res Ther. 2020 May 1;22:99. doi: 10.1186/s13075-020-2145-y (PMC7195789; doi:10.1186/s13075-020-2145-y)
Supplement: Supplementary file 4 — Additional file 4: Supplementary 4 Primer sequences of related genes for reverse transcription quantitative polymerase chain reaction. [file 13075_2020_2145_MOESM4_ESM.docx]

**Supplementary 4** **Primer sequences of related genes for reverse transcription quantitative polymerase chain reaction.**

| Gene | Primer sequence |
| --- | --- |
| miR-21-5p (forward) | 5’- CTTACTTCTCTGTGTGATTTCTGTG -3’ |
| miR-21-5p (reverse) | 5’- ACAACCTTTCCAAAATCCATGAGGC -3’ |
| Spry1 (forward) | 5’-ATAGGAGTGGACTGTGAA-3’ |
| Spry1 (reverse) | 5’-CCGTGTTCTAAGGAGTAG-3’ |
| U6 (forward) | 5’-TCCGATCGTGAAGCGTTC-3’ |
| U6 (reverse) | 5’-GTGCAGGGTCCGAGGT-3’ |
| β-actin (forward) | 5’-CTACCGTAAAGACCTCTATGC-3’ |
| β-actin (reverse) | 5’-GGAGCCAGAGCAGTAATCTC-3’ |
| VEGF (forward) | 5’-GGAGGGCAGAATCATCACG-3’ |
| VEGF (reverse) | 5’-TGGAAGATGTCCACCAGGG-3’ |
| MMP-13 (forward) | 5’-ATGGTCCAGGCGATGAAGAC-3’ |
| MMP-13 (reverse) | 5’-ACCCAGCCCTATCCCTTGAT-3’ |
| ADAMTS5 (forward) | 5’-CATAGAGAACACGCAGAG-3’ |
| ADAMTS5 (reverse) | 5’-CCAGCCAATACAGAACAT-3’ |
| ACAN (forward) | 5’-CAGTAGTGCGGACATTAG-3’ |
| ACAN (reverse) | 5’-AGAGGACAGTTCATTAAGC-3’ |
| COL-II (forward) | 5’-ACCGTTCTATTCCTCAGT-3’ |
| COL-II (reverse) | 5’-ACAGAGATGTAGCACCTT-3’ |
| IL-1β(forward) | 5’-ACGGACCCCAAAAGATGAAG-3’ |
| IL-1β(reverse) | 5’-TTCTCCACAGCCACAATGAG-3’ |

|  |  |
| --- | --- |
