# Supplementary material for: MiR-21-5p regulates extracellular matrix degradation and angiogenesis in TMJOA by targeting Spry1
Source: Arthritis Res Ther. 2020 May 1;22:99. doi: 10.1186/s13075-020-2145-y (PMC7195789; doi:10.1186/s13075-020-2145-y)
Supplement: Supplementary file 5 — Additional file 5: Supplementary 5 To verify that U0126 can significantly inhibit the activation of p-ERK1/2, the expression of p-ERK protein was detected. [file 13075_2020_2145_MOESM5_ESM.docx]

**Supplementary 5**

To verify that U0126 can significantly inhibit the activation of p-ERK1/2, the expression of p-ERK protein was detected.

**
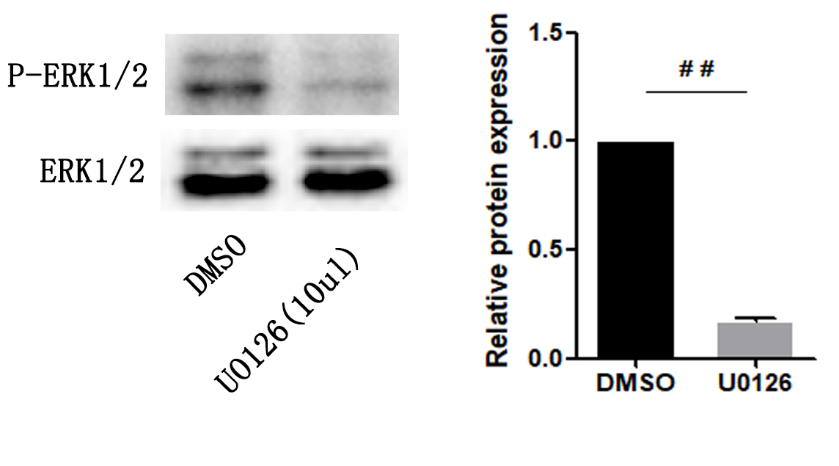
**

Data are represented as the means±standard deviation (n = 3) . Hash (**^##^**): compared with the DMSO group. **^##^**P < 0.05.
